# Antioxidant, Anti-Inflammatory, and Anticancer Activities of Five Citrus Peel Essential Oils

**DOI:** 10.3390/antiox13121562

**Published:** 2024-12-19

**Authors:** Yurong Li, Wenji Li, Zimao Ye, Chen Ji, Zhiqin Zhou

**Affiliations:** 1 Key Laboratory of Agricultural Biosafety and Green Production of Upper Yangtze River, Ministry of Education, College of Horticulture and Landscape Architecture, Southwest University, Beibei District, Chongqing 400715, China; yellor326815@163.com (Y.L.); yezimao@email.swu.edu.cn (Z.Y.); 1834606465@163.com (C.J.); 2 School of Design, Chongqing Industry Polytechnic College, Chongqing 401120, China; liwj@cqipc.edu.cn; 3 The Southwest Institute of Fruits Nutrition, Banan District, Chongqing 400054, China

**Keywords:** citrus, essential oil, antioxidants, anti-inflammatory, anticancer

## Abstract

Citrus peel essential oil (CPEO) is favored by people for its aromatic scent, while also possessing numerous bioactive compounds that are advantageous to human health. This study evaluated the antioxidant, anti-inflammatory, and anticancer activities of CPEOs through cell experiments. The results showed that CPEOs could increase the activity of the antioxidant enzyme system and nonenzymatic defence system in H_2_O_2_-treated RAW 264.7 cells by reducing cellular lipid peroxidation. CPEOs also reduced the nitric oxide production induced by lipopolysaccharide treatment in RAW 264.7 cells while decreasing proinflammatory cytokines expression and increasing anti-inflammatory cytokine expression. Wound healing assays, flow cytometry, and quantitative real-time fluorescent quantitative PCR (qRT-PCR) revealed that CPEOs could induce apoptosis in U87 cells through the mitochondrial apoptotic pathway. These findings indicate that CPEOs possess excellent antioxidant, anti-inflammatory, and anticancer activity potential, making them suitable for use in functional antioxidant and anti-inflammatory foods and nutritional health products.

## 1. Introduction

Citrus is an important fruit crop worldwide, being a significant component of daily human diets and consumed worldwide either fresh or in the form of juice. The global production of citrus fruits is approximately 158.5 million metric tons [1]. However, citrus processing generates a substantial amount of solid waste, with the global citrus processing industry producing over 10 million tons of waste annually [2]. The waste remaining after processing (peels, pulp, and seeds) is often only used as animal feed or is even discarded as trash, resulting in significant waste and increasing the risk of environmental pollution [3]. Within the industry, the treatment and reuse of citrus processing waste have always been considered key issues related to environmentally friendly ecological policies [4]. The peels of citrus fruits harbor numerous valuable components, including essential oils, polyphenols, vitamins, and dietary fiber [5]. The peels are the most important raw material for extracting citrus peel essential oils (CPEOs), which are in high demand globally and have broad market prospects, with sales in the international market reaching USD 500 billion [6]. Therefore, the scientific reuse of citrus processing waste can effectively address waste disposal issues and further increase the economic value of citrus.

CPEOs are mainly composed of hydrocarbons, phenols, alcohols, aldehydes, and other components and are natural, volatile, and complex plant secondary metabolites [3]. CPEOs are classified and generally recognized as safe (GRAS) by the United States Food and Drug Administration (FDA) [7]. CPEOs possess a delightful aroma and, in China, are permitted as natural food flavourings for addition to everyday edible products [8]. Owing to their rich content of bioactive compounds, CPEOs exhibit antioxidant, antibacterial, anti-inflammatory, anticancer, and cytoprotective activities, providing numerous health benefits to humans [9,10]. CPEOs are also used as natural food additives [11]. According to the Federal Register [12], the FDA has stated that CPEOs demonstrate antioxidant, liver-protective (hepatoprotective), and kidney-protective (nephroprotective) properties in various pathological conditions and instances of drug-induced toxicity [9,13].

Natural antioxidants, such as CPEOs, are considered important dietary components that promote human health [14]. Natural antioxidants can exert antioxidant and health-promoting effects through various mechanisms [15]. Human cells are constantly exposed to highly reactive oxygen species (ROS) generated either exogenously or endogenously, and these compounds can lead to oxidative stress [16]. Oxidative stress essentially represents an imbalance between oxidation and antioxidants during cellular respiration and metabolism, and this imbalance can cause severe damage to biological systems [17]. Intracellular oxidative stress can lead to inflammation through mechanisms such as the activation of inflammasomes [18]. The chronic inflammation induced by oxidative stress is a crucial factor in the pathogenesis of many metabolic diseases, such as cancer, diabetes, and cardiovascular diseases [19,20]. With increasing understanding of oxidative stress and inflammation, as well as increased attention to metabolic diseases, people have begun to seek various ways to combat oxidation. Some synthetic antioxidants, such as butylated hydroxytoluene, propyl gallate, tert-butylhydroquinone, and butylated hydroxyanisole, play a certain role, but they pose potential toxicity and safety issues [14,21]. The global demand for foods rich in natural antioxidants is increasing [22], and foods containing natural antioxidants such as CPEOs are receiving increasing attention [1].

Studies have shown that some components derived from natural plants can induce cytotoxicity in various human cancer cells while being nontoxic to normal epithelial cells [23,24]. These natural plant components are particularly important for patients who cannot tolerate the extreme side effects of chemotherapy, thus demonstrating significant pharmaceutical potential [25]. Currently, approximately half of conventional chemical drugs originate from plants, with approximately 25% derived directly from plants and another 25% being chemically modified derivatives of plant products [26]. Glioblastoma multiforme (GBM) is one of the most aggressive and poorly prognostic types of gliomas [27,28]. Owing to the presence of the human blood-brain barrier (BBB), conventional chemotherapy drugs have difficulty exerting their effects on GBM. Despite the combination of immunotherapy and targeted therapy after surgery, the prognosis of GBM remains poor. Studies have shown that the average survival time for GBM patients after surgery is only 6–14 months [29]. CPEOs possess unique lipophilicity, which increases their cellular absorption rate. After CPEOs are administered via oral or nasal inhalation routes, they can enter the bloodstream and cross the BBB to exert inhibitory effects [30,31]. Currently, CPEOs have become important substances for the treatment of various types of malignant tumours [32].

In this study, the peels of five citrus species were selected as raw materials for the preparation of CPEO extracts. The antioxidant and anti-inflammatory activities of the five CPEOs were evaluated in RAW264.7 cells. Additionally, we investigated the effects of citrus essential oils (EOs) on the proliferation, migration, and cell cycle of U87 glioblastoma cells, and assessed their ability to reduce the potential growth of cancer cells. The aim of this study was to explore the health-promoting and anticancer effects of CPEOs from different cultivated varieties and to provide a theoretical basis for the development of CPEOs as raw materials for health supplements and food products.

## 2. Materials and Methods

### 2.1. Plant Materials

In this study, five different cultivated citrus materials were selected, as detailed in Table 1. More information about plant materials is in Table S1. The fruits were consistent in shape, size, and colour, without visual defects or damage, and were immediately delivered to the College of Horticulture and Landscape Architecture, Southwest University (Chongqing, China), which houses the Key Laboratory of Agricultural Biosafety and Green Production of the Upper Yangtze River (under the Ministry of Education). The citrus materials were soaked in ultrapure water to remove surface dust and impurities, air dried at 25 °C, and subsequently stored in a refrigerator at 4 °C. The content of citrus essential oil is expressed as the weight of essential oil/the weight of peel.

### 2.2. Extraction of CPEOs

In accordance with the methods of Yao et al. [33], in this study, CPEOs were extracted from the peels of five different citrus varieties via the steam distillation method. First, 200 g of cleaned and crushed peels were added to a round-bottom flask. Then, 12 g of sodium chloride and 800 g of deionized water were added, and the extraction was performed through steam distillation. After filtration, the CPEO was dried with 0.3% anhydrous sodium sulphate. The collected citrus CPEOs were sealed and stored at −80 °C for future use. The volatile components of the CPEO were analysed by gas chromatography-mass spectrometry (GC-MS) with reference to the method described by Yao et al. [33].

### 2.3. Cell Culture and Reagents

RAW264.7 cells and U87 cells (human glioblastoma cells) were purchased from Procell Life Science & Technology Co., Ltd. (Wuhan, China). RAW 264.7 cells were cultured in Dulbecco’s modified Eagle’s medium (DMEM) (Procell, Wuhan, China) supplemented with 10% foetal bovine serum (FBS) (BI, Montevideo, Uruguay, South America) and 1% penicillin-streptomycin. U87 cells were grown in minimum essential medium (MEM) supplemented with 10% FBS and 1% penicillin-streptomycin. The cells were maintained in a humidified incubator at 37 °C with 5% CO_2_.

### 2.4. Cell Viability Assay

RAW264.7 and U87 cells were cultured in 96-well plates for 24 h at a density of 5 × 10^3^ cells per well. After 24 h of incubation, the medium was replaced with fresh medium containing different concentrations of CPEOs as the treatment medium, and fresh medium containing 0.5% DMSO served as the control medium (CK). Mitoxantrone (Sigma-Aldrich, Shanghai, China) was used as a positive control for U87 cells (Figure S1). After 24 h of CPEO treatment, cell toxicity was assessed via a Cell Counting Kit-8 (CCK8) from Biosharp (Hefei, China). The absorbance values were measured at a wavelength of 450 nm via a microplate reader, and the percentage of cell viability was calculated for both the control group and the different treatment groups. The experiment was independently repeated three times.

### 2.5. Evaluation of the Antioxidant Activity of CPEOs

#### 2.5.1. Establishment of an Oxidative Stress Model in RAW264.7 Cells

RAW264.7 cells were subjected to oxidative stress induction with slight modifications to the method described by Zhou et al. [34]. Hydrogen peroxide (H_2_O_2_) was purchased from Chongqing Chuandong Chemical Co., Ltd. (Chongqing, China). First, RAW264.7 cells were seeded into 96-well plates and allowed to grow for 24 h. Ascorbic Acid (AsA) was used as a positive control at a concentration of 25 µg/mL (Sigma-Aldrich, Shanghai, China). The treatment group was exposed to CPEOs for 24 h. Afterwards, the cells were washed twice with warm PBS. In addition to the control group, H_2_O_2_ diluted to 0.03% with complete medium was added for 2 h to induce oxidative stress. Subsequently, cell viability was determined via a Cell Counting Kit-8 (CCK8) assay. The experiment was independently repeated three times.

#### 2.5.2. Measurement of Malondialdehyde and Glutathione Concentrations and Antioxidant Enzyme Activities in RAW264.7 Cells

The malondialdehyde (MDA) and glutathione (GSH) concentration measurement kits, the enzyme activity detection kits for superoxide dismutase (SOD), catalase (CAT), glutathione reductase (GR), and the bicinchoninic acid (BCA) protein detection kit were purchased from Solarbio Life Science and Technology Ltd. (Beijing, China). RAW264.7 cells were first seeded into 6-well plates and preincubated for 24 h. Oxidative stress induction was then performed according to the methods described in Section 2.5.1. Afterwards, the cells were collected and resuspended in extraction solution. The activity of the SOD enzyme was assessed by measuring the absorbance value at 450 nm with a microplate reader. The activities of GR and CAT were determined via a UV-spectrophotometric method, with absorbance readings taken at 340 nm and 240 nm, respectively, with a microplate reader. The concentration of MDA was measured by determining the absorbance values at 532 nm and 600 nm with a microplate reader. The concentration of GSH was measured with the corresponding kit, with absorbance readings taken at 412 nm. The protein concentration was measured with the BCA kit. The experiment was independently repeated three times.

### 2.6. Assessment of the Anti-Inflammatory Activities of CPEOs

#### 2.6.1. Establishment of an Inflammatory Model Using RAW264.7 Cells

We induced inflammation in RAW264.7 cells by using lipopolysaccharide (LPS). LPS was purchased from Nanjing Herbal Biotechnology Co., Ltd. (Nanjing, China). First, RAW264.7 cells were seeded into 96-well plates and allowed to grow for 24 h. The original medium was aspirated, and then, 3 µg/mL LPS diluted with complete medium was added to induce inflammation for 4 h. The medium was aspirated again, and the cells were washed twice with warm PBS. The treatment group subsequently received CPEOs, which were administered for 24 h. Dexamethasone (DEX), a standard anti-inflammatory agent, was used as a positive control at a concentration of 5 µg/mL (Sigma-Aldrich, Shanghai, China). The experiment was independently repeated three times.

#### 2.6.2. Measurement of the Nitric Oxide Concentration in RAW264.7 Cells

The nitric oxide (NO) concentration in the cell culture medium was measured with an NO detection kit (Beyotime, Shanghai, China). After allowing the GriessA and B solutions to reach room temperature, 50 μL of the supernatant from each well of the control and treatment groups described in Section 2.6.1 was transferred to a new 96-well plate. Next, the GriessA and B solutions were mixed in equal volumes, and then 100 μL of the mixed solution was added to each well of a 96-well plate containing the supernatant samples. The absorbance at 540 nm was measured with a microplate reader. The NO concentration in the samples was calculated on the basis of the standard curve. The experiment was independently repeated three times.

#### 2.6.3. Measurement of the Relative Expression Levels of Inflammatory Cytokines in RAW264.7 Cells

The cells were collected, and 500 µL of cell lysis buffer (TIANGEN, Beijing, China) was added. The Total RNA Extraction Kit was purchased from TIANGEN (Beijing, China), while the Reverse Transcription Kit and SYBR Green Master Mix were purchased from Vazyme (Nanjing, China). RNA was extracted from the cells according to the manufacturer’s instructions. The isolated total RNA was subsequently reverse transcribed into cDNA following the manufacturer’s instructions. Quantitative real-time fluorescent quantitative PCR (qRT-PCR) analysis was performed on the Bio-Rad CFX96 system using gene-specific primers. For the quantification of the PCR products, SYBR Green Master Mix was used according to the manufacturer’s instructions. All the data were normalized to the GAPDH expression level. The primer sequences are provided in Supplementary Table S2.

### 2.7. Evaluation of the In Vitro Antitumour Activity of CPEOs

#### 2.7.1. Proliferation Analysis of U87 Cells

The antiproliferative effect of CPEOs was analysed with the EdU Cell Proliferation Kit (Beyotime, Shanghai, China), Triton X-100 (Beyotime, Shanghai, China), and 4% paraformaldehyde fixative (Beyotime, Shanghai, China). U87 cells were treated with CPEOs for 24 h, after which the medium was aspirated. The cells were thoroughly washed three times with PBS. Next, the cells were placed in an EdU (10 µM) working solution and incubated in the dark at 37 °C for 2 h. After incubation, the cells were washed three times with PBS and fixed with 4% paraformaldehyde for 15 min. The cells were subsequently permeabilized with 0.3% Triton X-100 for 15 min and washed with PBS three times. Finally, the cells were incubated with Hoechst 33342 for 10 min. Staining was performed according to the kit’s instructions, and images were captured with a fluorescence microscope (Olympus U-HGLGPS, Shanghai, China). The experiment was independently repeated three times.

#### 2.7.2. U87 Cell Wound Healing Assay

U87 cells were seeded at 1 × 10^6^ cells/well in a 6-well plate and cultured at 37 °C for 24 h in a 5% CO_2_ environment. A sterile 200 μL pipette was used to cross-scratch the cell monolayer, which was then washed with PBS three times to remove floating cells. The cells were then treated with CPEOs. At 0 h and 24 h, the treated cells were imaged with an inverted fluorescence microscope (Olympus U-HGLGPS, Suzhou, China) and photographed. The experiment was independently repeated three times. ImageJ software (version 1.4.3.67) was used to analyse mobility. The formula for calculating the cell scratch healing rate was (W 0 h − W 24 h)/W 0 h × 100%.

#### 2.7.3. Analysis of the U87 Cell Cycle and Apoptosis

Cell cycle and apoptosis were evaluated with the Cell Cycle Analysis Kit (PI) (Biosharp, Hefei, China) and the Annexin V-FITC/PI Apoptosis Kit (Elabscience, Wuhan, China). After U87 cells were treated with CPEOs for 24 h, the cells were harvested, washed twice with PBS at 4 °C, and then resuspended in 1 × Annexin V binding buffer. Subsequently, 2.5 μL of Annexin V-FITC and 2.5 µL of PI were added, and the cells were stained in the dark for 15 min for apoptosis analysis. The cells intended for cell cycle evaluation were fixed in 70% ethanol at 4 °C for 12 h. Then, 0.5 mL of PI was added, and the cells were stained in the dark at 37 °C for 30 min. The distribution of cells in different phases of the cell cycle and apoptosis was determined via flow cytometry (BD, PerkinElmer, Shelton, CT, USA). A total of 2 × 10^4^ cells were counted per sample. The experiments were independently repeated three times.

#### 2.7.4. Analysis of the Mitochondrial Membrane Potential in U87 Cells

Mitochondrial membrane potential (MMP) was evaluated with the Enhanced Mitochondrial Membrane Potential Assay Kit with JC-1 (Solarbio Life Science and Technology Ltd., Beijing, China). After U87 cells were treated with CPEOs for 24 h, the original culture medium was aspirated, and the cells were washed twice. The cells were then incubated with JC-1 solution at 37 °C for 20 min. Next, the cells were washed twice with JC-1 buffer solution. The fluorescence signals were detected with a fluorescence microscope (Olympus U-HGLGPS, Shanghai, China), and the fluorescence intensity was measured with a multifunctional microplate reader. The experiments were independently repeated three times.

#### 2.7.5. Measurement of the Relative Expression Levels of Apoptosis-Related Genes in U87 Cells

The method is the same as that described in Section 2.6.3. The primer sequences are provided in Supplementary Table S2.

### 2.8. Statistical Analysis

All the experiments were repeated three times. The results are presented as the mean ± standard deviation (mean ± SD). Statistical graphs and data analysis were performed via OriginPro 2021 software. Analysis of variance was performed to analyse the significance of differences among the different groups using Duncan’s multiple range test (*p* < 0.05) following one-way ANOVA.

## 3. Results

### 3.1. Effect of CPEOs on the Cell Inhibition Rate of RAW 264.7 and U87 Cells

**Figure 1:**
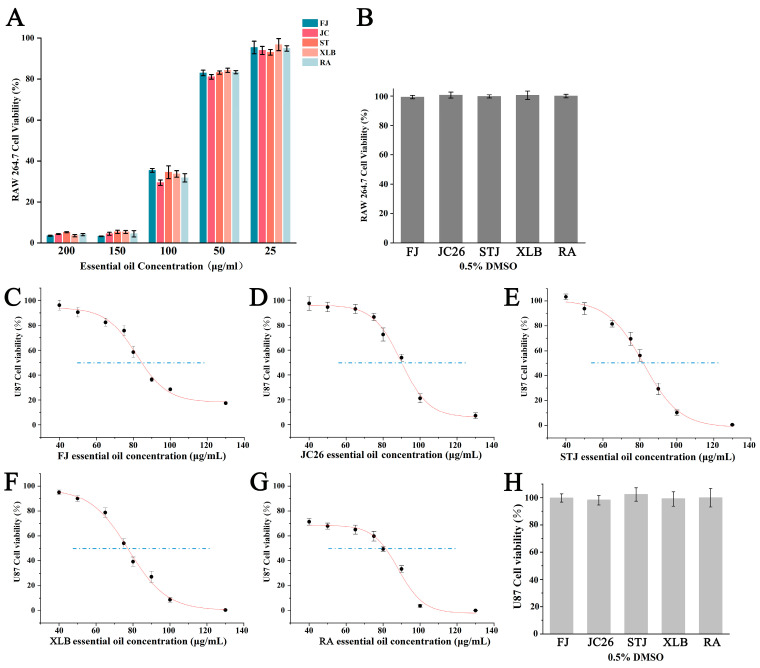
The effect of five CPEOs on the cell viability of RAW264.7 cells and U87 cells. (**A**) The changes in cell viability after treating RAW264.7 cells with different concentrations of FJ, JC26, STJ, XLB, and RA EOs for 24 h. (**B**) Treatment with 0.5% DMSO did not affect the viability of RAW264.7 cells. (**C**–**G**) The changes in cell viability after treating U87 cells with different concentrations of FJ, JC26, STJ, XLB, and RA EOs for 24 h. (**H**) Treatment with 0.5% DMSO did not affect the viability of U87 cells. The data represent the mean ± SD. The blue dashed line indicated a cell viability rate of 50%.

A CCK8 kit (Biosharp, Hefei, China) was used to evaluate the cell inhibition rate of the EOs of five citrus varieties on RAW264.7 cells and U87 cells. The viability of RAW264.7 and U87 cells after incubation with different varieties of CPEOs for 24 h is shown in Figure 1. The control group was treated with 0.5% DMSO (labelled CK), and the cell viability was unaffected (Figure 1B,H).

The results for RAW264.7 cells showed that at a concentration of 100 µg/mL for the five CPEOs, the survival rate of RAW264.7 cells was less than 40%. At concentrations of 25 and 50 µg/mL, the survival rates were greater than 80% (Figure 1A). Therefore, 50 µg/mL was selected as the concentration for subsequent antioxidant and anti-inflammatory experiments.

The viability of U87 cells was significantly reduced in a dose-dependent manner (Figure 1C–G). The IC_50_ values for the five EOs of FJ, JC26, STJ, XLB, and RA were 84.81 ± 1.01 μg/mL, 89.67 ± 0.87 μg/mL, 82.05 ± 0.95 μg/mL, 81.02 ± 0.93 μg/mL, and 81.02 μg/mL, respectively. The IC_50_ values of XLB and RA EOs were lower than those of the other EOs, indicating stronger cell inhibitory effects at lower concentrations (Table 2). Therefore, in subsequent experiments, the cells were treated with XLB and RA EOs at concentrations of 40 and 80 μg/mL (labelled XLB40, XLB80; RA40, RA80).

### 3.2. Antioxidant Activity of CPEOs

#### 3.2.1. Effects of CPEOs on the Viability of H_2_O_2_-Treated RAW264.7 Cells

**Figure 2:**
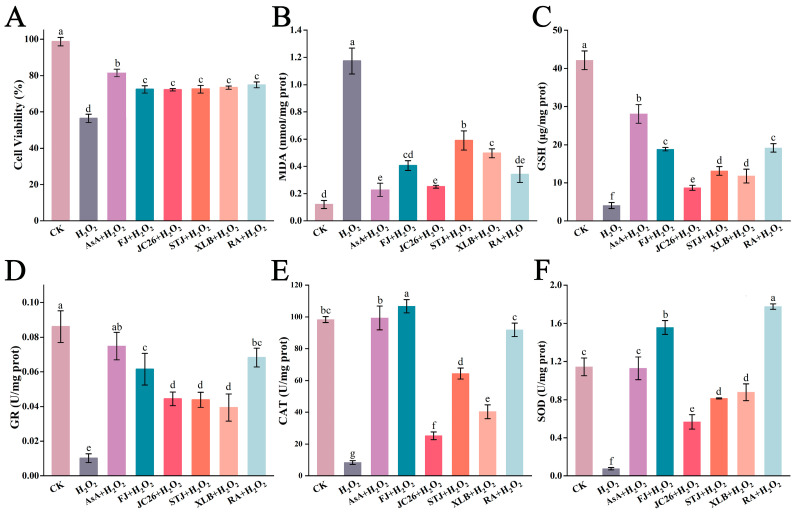
T The effect of CPEOs on cell viability (**A**), MDA (**B**), GSH (**C**), GR (**D**), CAT (**E**), and SOD (**F**) in RAW264.7 cells after induction with 0.03% H_2_O_2_ for 2 h. CK was treated with 0.5% DMSO. The concentration of AsA in the positive control was 25 µg/mL. The concentration of the five CPEOs was 50 μg/mL. The data represent the mean ± SD. “pro” represents protein. Values followed by different superscripts (a–g) are significantly different (*p* < 0.05).

H_2_O_2_ can rapidly penetrate membranes and induce oxidative stress, which may cause damage to cells. Figure 2A shows that, compared with that in the CK group, the cell survival rate in the H_2_O_2_ group was 0.57-fold lower. However, after the addition of 50 µg/mL FJ, JC26, STJ, XLB, or RA, the survival rates of the RAW264.7 cells increased by 1.28-fold, 1.28-fold, 1.28-fold, 1.30-fold, or 1.33-fold, respectively. Thus, CPEOs effectively protected RAW264.7 cells from H_2_O_2_-induced damage.

#### 3.2.2. Effects of CPEOs on MDA and GSH Concentrations in H_2_O_2_-Treated RAW264.7 Cells

Figure 2B shows that, compared with that in the CK group, the MDA concentration in the H_2_O_2_ group increased 9.47-fold. However, after the addition of 50 µg/mL FJ, JC26, STJ, XLB, and RA EOs, the MDA concentration in H_2_O_2_-treated RAW 264.7 cells was reduced by 0.35-fold, 0.21-fold, 0.49-fold, 0.39-fold, and 0.30-fold, respectively, compared with that in the H_2_O_2_ group. Therefore, treatment with CPEOs significantly decreased the MDA concentrations in RAW264.7 cells subjected to oxidative stress, reducing the degree of lipid peroxidation in the cell membrane. Among them, RA and JC26 EOs had the greatest effects.

Figure 2C shows that the GSH concentration in RAW264.7 cells in the H_2_O_2_ group significantly decreased from 42.14 ± 2.45 μg/mg prot in the CK group to 4.06 ± 0.79 μg/mg prot. The addition of 50 μg/mL of the FJ, JC26, STJ, XLB, and RA EOs significantly increased the GSH concentrations by 4.64-fold, 2.14-fold, 3.23-fold, 2.90-fold, and 4.72-fold, respectively. These results indicate that these CPEOs can increase GSH concentrations in RAW264.7 cells under oxidative stress conditions, thereby increasing their cellular antioxidant capacity. Among them, RA and FJ EOs exhibited the most potent effects.

#### 3.2.3. Effects of CPEOs on the Activities of GR, CAT, and SOD in H_2_O_2_-Treated RAW264.7 Cells

CPEOs significantly restored the activity of GR in H_2_O_2_-treated RAW264.7 cells (Figure 2D). Compared with that in the CK group, the GR activity in the H_2_O_2_ group decreased by 8.43-fold. After the addition of 50 µg/mL FJ, JC26, STJ, XLB, or RA EOs, the GR activity increased by 6.02-fold, 4.35-fold, 4.30-fold, 3.86-fold, or 6.68-fold, respectively. Thus, CPEOs can significantly increase GR activity in RAW264.7 cells under oxidative stress conditions. Among them, RA and FJ EOs exhibited the most potent effects.

CPEOs significantly restored CAT activity in RAW264.7 cells (Figure 2E). The CAT activity in the CK group was 98.34 ± 1.87 U/mg prot, whereas it decreased to 8.45 ± 1.19 U/mg prot in the H_2_O_2_ group. After the addition of 50 µg/mL FJ, JC26, STJ, XLB, or RA EOs, the CAT activity increased by 12.64-fold, 2.98-fold, 7.51-fold, 4.78-fold, or 10.89-fold, respectively. CPEOs can have a significant effect on CAT activity in RAW264.7 cells under oxidative stress conditions, improving the cellular antioxidant defence mechanism. Among them, the FJ and RA EOs had the most potent effects.

The activity of SOD in the different groups is shown in Figure 2F. The SOD activity in the CK group was 1.07 ± 0.09 U/mg prot. Compared with that in the CK group, the SOD activity in the H_2_O_2_ group decreased by 12.73-fold. However, after the addition of 50 µg/mL FJ, JC26, STJ, XLB, and RA EOs, the SOD activity increased to 18.11-fold, 5.95-fold, 9.74-fold, 9.88-fold, and 21.04-fold greater than that of the H_2_O_2_ group, respectively. In summary, after 24 h of treatment with the five CPEOs, the SOD activity in RAW264.7 cells significantly increased. Among them, RA and FJ EOs exhibited the most potent effects.

### 3.3. Anti-Inflammatory Activities of CPEOs

#### 3.3.1. Effect of CPEOs on the NO Concentration in RAW264.7 Cells

**Figure 3:**
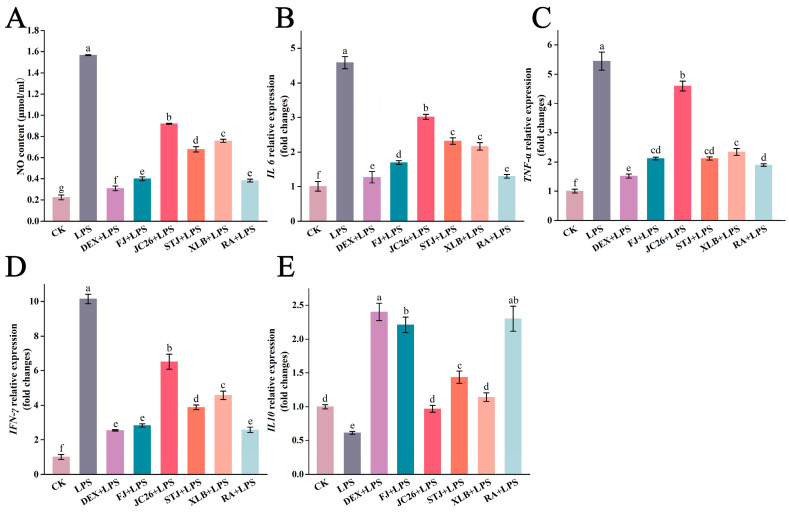
The effect of CPEOs on the production of NO (**A**), *IL-6* (**B**), *TNF-α* (**C**), *IFN-γ* (**D**), and *IL-10* (**E**) in RAW264.7 cells after induction with 3 μg/mL LPS for 4 h. CK was treated with 0.5% DMSO. The concentration of DEX in the positive control was 5 µg/mL. The concentration of the five CPEOs was 50 μg/mL. The data represent the mean ± SD. Values followed by different superscripts (a–g) are significantly different (*p* < 0.05).

The experimental results indicate that all five CPEOs inhibited LPS-induced NO production (Figure 3A). The NO concentration in the CK group RAW264.7 cells was 0.22 ± 0.02 µmol/mL. After LPS stimulation, the NO concentration increased significantly to 1.57 ± 0.01 µmol/mL, a 6.99-fold increase compared with the CK group. When 50 µg/mL FJ, JC26, STJ, XLB, or RA Eos were added, the NO concentrations decreased 0.25-fold, 0.59-fold, 0.43-fold, 0.48-fold, and 0.24-fold that of the LPS group, respectively. Therefore, with the addition of FJ and RA EOs, the NO concentration returned to near-normal levels, suggesting that these CPEOs can effectively ameliorate the increase in NO concentration caused by cellular inflammation.

#### 3.3.2. Effect of CPEOs on the Relative Expression Levels of Inflammation-Related Genes in RAW264.7 Cells

We determined that after LPS treatment, the gene expression levels of the proinflammatory cytokines *IL-6*, *TNF-α*, and *IFN-γ* in the cells significantly increased compared with those in the CK group. In contrast, the gene expression level of the anti-inflammatory cytokine IL-10 significantly decreased compared with the CK group. However, treatment with 50 µg/mL FJ, JC26, STJ, XLB, or RA EOs effectively reduced *IL-6*, *TNF-α*, *IFN-γ* expression levels (Figure 3B–D). Compared with that in the CK group, the *IL-6* expression level in the LPS group increased by 4.56-fold. Nevertheless, after treatment with the five CPEOs, the *IL-6* expression levels decreased by 0.37-fold, 0.66-fold, 0.51-fold, 0.47-fold, and 0.28-fold, respectively, compared with those in the LPS group. The *TNF-α* expression level in the LPS group was 5.44-fold higher than that in the CK group. The *TNF-α* expression levels in the groups treated with FJ, JC26, STJ, XLB, or RA were 0.39-fold, 0.84-fold, 0.39-fold, 0.43-fold, and 0.35-fold greater than those in the LPS group, respectively. Compared with that in the CK group, the relative expression level of *IFN-γ* in the LPS group increased 10.09-fold. However, in the groups treated with FJ, JC26, STJ, XLB, or RA, the relative expression levels of *IFN-γ* decreased by 0.28-fold, 0.64-fold, 0.38-fold, 0.45-fold, and 0.25-fold, respectively, compared with those in the LPS group. Compared with that in the CK group, the relative expression level of *IL-10* in the LPS group decreased 0.61-fold (Figure 3E). The *IL-10* expression levels in the groups treated with FJ, JC26, STJ, XLB, or RA were 3.61-fold, 1.58-fold, 2.35-fold, 1.87-fold, and 3.76-fold greater than those in the LPS group, respectively.

### 3.4. Anticancer Activity of CPEOs

#### 3.4.1. Effect of CPEOs on the Proliferation and Migration of U87 Cells

**Figure 4:**
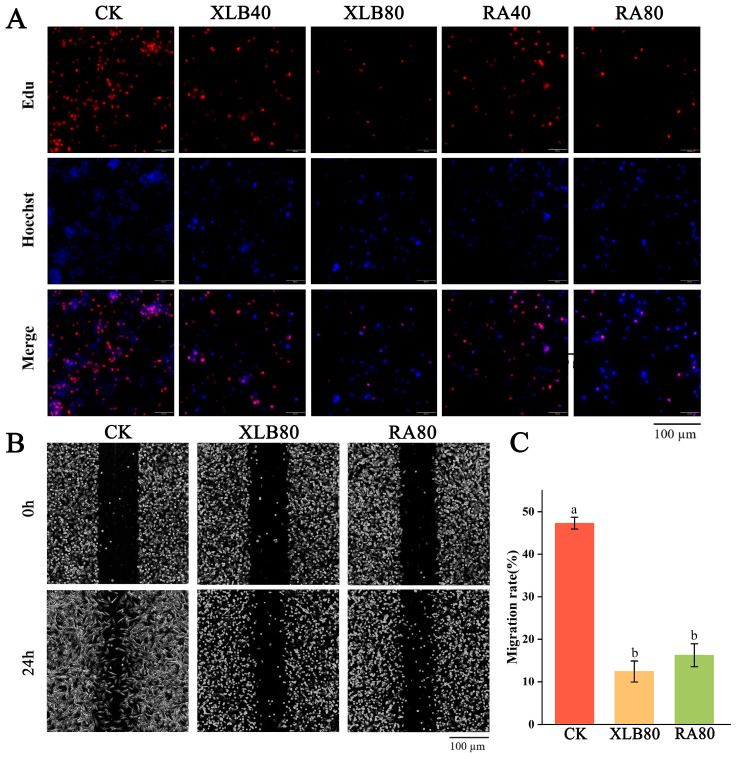
Effect of the CPEOs on proliferation and migration in U87 cells. (**A**) XLB and RA EOs can inhibit cell proliferation. Proliferating cells and cell nuclei were stained with BeyoClick™ EdU-555 kit and Hoechst 33342. (**B**) The cell migration ability of U87 cells was determined using the wound healing assay, where the cells were treated with XLB and RA EOs in serum-free medium for 24 h. (**C**) Statistical data of migration rate (%). The concentrations of XLB40 and RA40 were 40 µg/mL, while those of XLB80 and RA80 were 80 µg/mL. CK was treated with 0.5% DMSO. The data represent the mean ± SD. Values followed by different superscripts (a,b) are significantly different (*p* < 0.05).

As shown in Figure 4A, the cells emitting red fluorescence were in the proliferation phase. When treated with XLB and RA EOs at a concentration of 40 µg/mL, the number of cells emitting red fluorescence significantly decreased compared with that in the control group. At a concentration of 80 µg/mL for XLB and RA EOs, the number of proliferating cells was markedly reduced. DNA synthesis in the cells was significantly inhibited in a dose-dependent manner.

The results of the in vitro wound healing assay are shown in Figure 4B. Compared with that of the CK group, the confluence of U87 cells was significantly reduced after treatment with 80 µg/mL XLB and RA EOs for 24 h. The percentage of migrated cells in the CK group at 24 h was 47.32 ± 1.43%, whereas the percentage in the XLB80 group was 12.43 ± 2.47%, and the percentage in the RA80 treatment group was 16.24 ± 2.69% (Figure 4C). Therefore, XLB and RA EOs can inhibit cell migration.

#### 3.4.2. Effects of CPEOs on the Cell Cycle of U87 Cells

**Figure 5:**
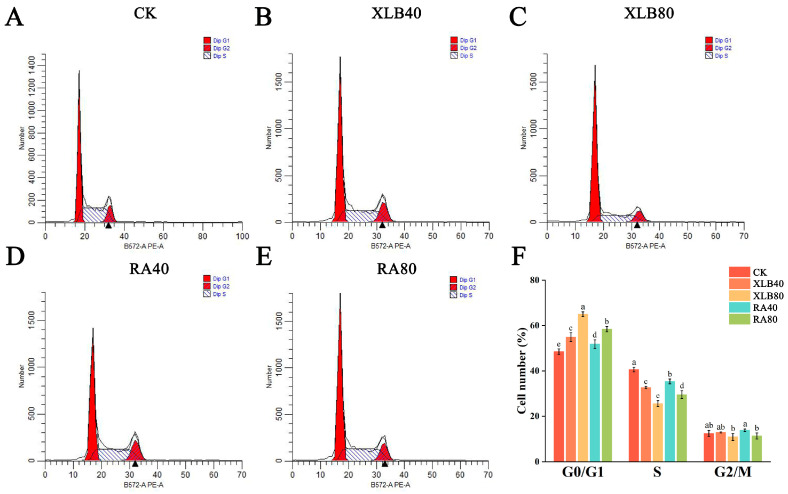
Effects of the CPEOs on the U87 cells cycle. (**A**–**E**) Determination of cell cycle distribution by flow cytometry. (**F**) The proportions of cell populations in G0/G1, S, and G2/M phases after treatment with XLB and RA EOs. The concentrations of XLB40 and RA40 were 40 µg/mL, while those of XLB80 and RA80 were 80 µg/mL. CK was treated with 0.5% DMSO. The data are presented as mean ± SD. Values followed by different superscripts (a–e) are significantly different (*p* < 0.05).

As shown in Figure 5, compared with that in the CK group, the distribution of U87 cells in different phases was significantly altered after intervention with the two types of CPEOs for 24 h. With increasing concentrations of XLB and RA EOs, the relative percentage of U87 cells in the G0/G1 phase gradually increased. The relative percentage of cells in the G0/G1 phase under XLB80 treatment was 64.99 ± 0.86%, and that under RA80 treatment was 58.40 ± 0.97%. The percentage of cells in the S phase was significantly reduced. These results indicate that CPEOs inhibit cell proliferation by blocking cell cycle progression at the G0/G1 phase.

#### 3.4.3. Effect of CPEOs on the Apoptosis of U87 Cells

**Figure 6:**
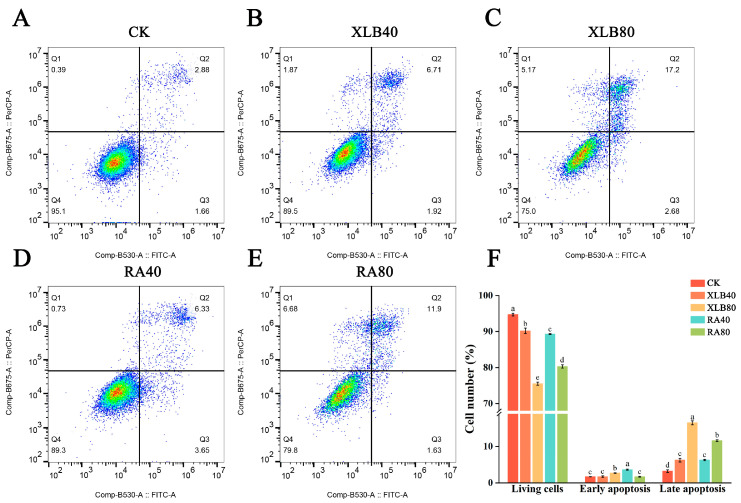
Effects of the CPEOs on U87 cell apoptosis. (**A**–**E**) Apoptotic cells were assessed by flow cytometry after Annexin V-FITC and PI staining. Q1 represents necrotic cells; Q2 represents late apoptotic cells; Q3 represents early apoptotic cells; Q4 represents viable cells. (**F**) Living cells, early and late apoptotic cell rates. The concentrations of XLB40 and RA40 were 40 µg/mL, while those of XLB80 and RA80 were 80 µg/mL. CK was treated with 0.5% DMSO. The data are presented as mean ± SD. Values followed by different superscripts (a–e) are significantly different (*p* < 0.05).

As shown in Figure 6, the apoptosis rate of U87 cells after intervention with the two types of CPEOs for 24 h was significantly greater than that of the CK group. The proportion of viable cells decreased from 94.7 ± 0.36% in the CK group to 75.5 ± 0.44% in the XLB80 group and 80.3 ± 0.46% in the RA80 group. The number of apoptotic cells increased from 4.92 ± 0.3% in the CK group to 19.25 ± 0.55% in the XLB80 group and 13.27 ± 0.24% in the RA80 group. The percentage of apoptotic cells, especially late apoptotic cells, increased significantly with increasing drug concentration. These results indicate that CPEOs can induce apoptosis in U87 cells.

#### 3.4.4. Effect of CPEOs on the MMP in U87 Cells

**Figure 7:**
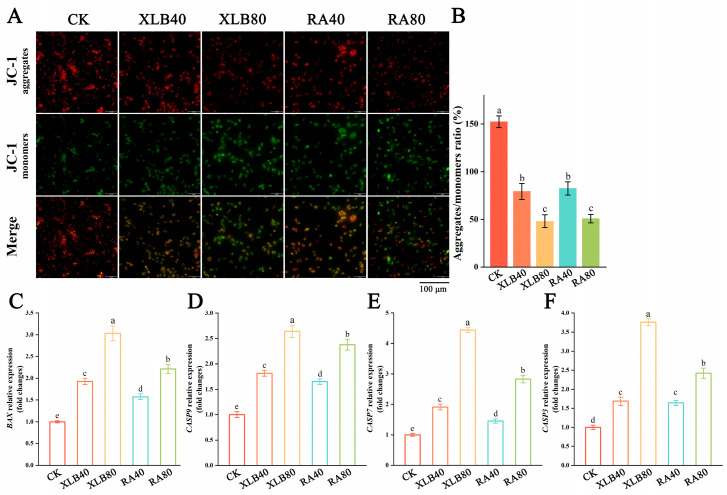
Effect of the CPEOs on the MMP and the relative expression levels of apoptosis-related genes in U87 cells. (**A**) Fluorescence images of U87 incubated with JC-1 after treatment with XLB and RA EOs. (**B**) MMP quantified by measuring green fluorescence intensity and red fluorescence intensity. (**C**) *BAX*; (**D**) *Caspase-9*; (**E**) *Caspase-7*; (**F**) *Caspase-3*. The concentrations of XLB40 and RA40 were 40 µg/mL, while those of XLB80 and RA80 were 80 µg/mL. CK was treated with 0.5% DMSO. The data are presented as mean ± SD. Values followed by different superscripts (a–e) are significantly different (*p* < 0.05).

A decrease in the red/green fluorescence intensity ratio in cancer cells stained with JC-1 dye indicates mitochondrial depolarization, leading to apoptosis. Healthy U87 cells exhibited red fluorescence, whereas cells treated with XLB and RA EOs for 24 h presented an increase in green fluorescence intensity and a decrease in red fluorescence intensity, indicating significant disruption of the mitochondrial membrane potential (Figure 7A). As shown in Figure 7B, the ratio of the JC-1 monomer to the JC-1 polymer increased with increasing drug concentration. Compared with those of the CK group, the MMPs of the XLB40, XLB80, RA40, and RA80 groups decreased to 0.79 ± 0.08, 0.47 ± 0.07, 0.82 ± 0.07, and 0.51 ± 0.04, respectively. Therefore, CPEOs reduce the mitochondrial membrane potential of U87 cells in a dose-dependent manner.

#### 3.4.5. Effect of CPEOs on the Relative Expression Levels of Apoptosis-Related Genes in U87 Cells

To further evaluate the effect of CPEOs on apoptosis in U87 cells, qRT-PCR was used to measure the expression levels of apoptosis-related genes. In our study, significant changes in the expression levels of the proapoptotic gene *BAX* were observed in U87 cells treated with XLB and RA EOs (Figure 7C). Compared with that in the CK group, the expression of the *BAX* gene was significantly upregulated by 1.93-fold, 3.03-fold, 1.58-fold, and 2.22-fold in the XLB40, XLB80, RA40, and RA80 groups, respectively. A significant increase in *BAX* expression indicates the induction of apoptosis through the activation of components of the caspase cascade, such as *caspase-9*, *caspase-7*, and *caspase-3*. As shown in Figure 7D, compared with that in the CK group, the expression level of the *caspase-9* gene increased in a concentration-dependent manner by 1.81-fold, 2.64-fold, 1.65-fold, and 2.38-fold in the XLB40, XLB80, RA40, and RA80 groups, respectively. In the XLB40 and RA40 groups, *caspase-7* expression was upregulated by 1.91-fold and 1.45-fold, respectively. The expression of the *caspase-7* gene was significantly upregulated by 1.32-fold and 1.41-fold in the XLB80 and RA80 groups, respectively (Figure 7E). Similarly, the expression level of *caspase-3* increased in a concentration-dependent manner, by 1.68-fold, 3.75-fold, 1.64-fold, and 2.42-fold, in the XLB40, XLB80, RA40, and RA80 groups, respectively (Figure 7F).

## 4. Discussion

Foods containing natural antioxidants are becoming increasingly popular because of their ability to neutralize and eliminate free radicals, thereby contributing to human health. CPEOs have become one of the most valuable botanical products used in drug and supplement therapeutic strategies [32]. CPEOs have garnered widespread attention because of their potent antioxidant and anti-inflammatory activities and have shown promise in preventing/inhibiting cancer [35]. In our study, all five types of CPEOs exhibited good antioxidant and anti-inflammatory activities, as well as cytotoxic effects on glioblastoma cells, indicating significant potential for development as natural food additives.

In China, the production and industrial consumption of oranges and grapefruits are huge, and the outer peels of oranges and grapefruits are thick and have high levels of EO, which is beneficial for the extraction of essential oils from citrus peels [1,6]. Kumquat is the only citrus variety whose peel and pulp can be eaten together [36]. Therefore, in the selection of materials for this study, we chose the peels of these five citrus species for the extraction of essential oils from citrus peels. In another study of ours [37], the chemical compositions of five citrus peel essential oil (CPEO) types were analysed via GC-MS and it was found that RA and STJ had relatively high amounts of D-limonene, while XLB and RA contained relatively high amounts of α-pinene, and FJ and RA had relatively high amounts of β-myrcene. D-limonene, a major compound in CPEOs with anticancer activity, was present in percentages ranging from 32% to 98% [38]. D-limonene is a monoterpenoid natural product renowned for its pleasant lemon aroma, is considered nontoxic and safe, and is often used as a flavouring agent in common foods [5]. D-limonene is considered to be the main anti-proliferative component in CPEO and has been extensively studied for its anticancer activity across many different cancer types [39]. Research has shown that the combination of D-limonene and docetaxel increases cytotoxic effects against prostate cancer cells but not against normal prostate epithelial cells [40]. In our study, all five CPEOs exhibited strong inhibitory effects on U87 cells. Thanks to its high D-limonene content, RA showed the strongest inhibitory effects and lower IC_50_ values. We speculated that this is the main factor for RA EO to exert its anticancer activity. On the one hand, some studies have reported that β-myrcene, a natural monoterpene, has demonstrated antiproliferative activity against human cervical cancer cells and human lung cancer cells [41]. We hypothesized that the good inhibitory effect of RA on U87 cells, in addition to its high content of D-limonene, may also benefit from its high content of β-myrcene.

While XLB is not a high-D-limonene-containing EO, it has a stronger inhibitory effect on cancer cells than does STJ, which has a higher D-limonene content, possibly because it has the highest content of α-pinene. α-Pinene is an important bicyclic double-bonded terpene hydrocarbon widely used in fragrances, antiviral agents, and antibacterial agents [42]. α-Pinene has anticancer effects on human ovarian cancer cell lines, hepatocellular carcinoma cell lines, and N2a neuroblastoma cells [43,44]. Additionally, α-pinene has protective effects on rat IEC-6 cells (intestinal epithelial cells), increasing the viability of IEC-6 cells [45]. In this study, when RAW263.7 cells were treated with the five CPEOs at a concentration of 100 µg/mL, the percentage of viable cells was greater than 30% with all the treatments. However, the viability of U87 cells significantly decreased under the same concentration treatment. This may be due to the faster division rate of cancer cells and their greater sensitivity to CPEOs compared to normal cells. This inhibitory effect on cancer cells increases with increasing CPEOs concentration. There is also evidence that the lipophilic monoterpenes (D-limonene and α-pinene) in CEPO may have a protective effect on normal cells [42,46], which may be another reason for this situation. In addition, multiple pieces of evidence indicated that the anticancer cell proliferation effect of the combination of D-limonene and monomer components in CPEO is significantly better than that of the monomer compounds [46,47]. A study by Palma et al. [4] revealed that the secondary components in citrus Eos synergize with D-limonene to exert a greater inhibitory effect on the human mammary tumour cell line MCF-7 than on the Chinese hamster ovary nontumour cell line AA8. In our study, the cytotoxicity of CPEOs may be related to the synergistic effect of different components, such as α-pinene and β-myrcene, which may enhance the effect of D-limonene. Therefore, among the five essential oils, XLB and RA have the best inhibitory effect on U87.

The human body has mechanisms to balance oxidative stress. However, due to factors such as unbalanced dietary patterns [48], environmental pollution [49], and unhealthy lifestyles [50], the oxidative stress system in the human body becomes imbalanced and triggers the accumulation of ROS, which may lead to cellular DNA damage, lipid peroxidation, and protein oxidation, and further trigger programmed cell death [48]. Therefore, exogenous supplementation is needed to balance the redox system. In this study, we found that CPEOs can maintain the redox homeostasis of RAW264.7 cells after oxidative stress induction, decrease the level of MDA, and increase cell survival rates. Current evidence suggests that major components in CPEOs, such as D-limonene, α-pinene, linalool, and myrcene, can significantly inhibit cellular lipid peroxidation and reduce the production of ROS [51,52,53]. Oxidative stress occurs when the production of ROS increases and the levels of antioxidant enzymatic defence systems and non-enzymatic defence systems (such as GSH) decrease [50]. In our study, it was observed that CPEOs can increase the activity of antioxidant enzymes (GR, CAT, and SOD), elevate GSH levels, and thereby reduce H_2_O_2_-induced oxidative stress. Existing evidence indicated that treatment with D-limonene and α-pinene can enhance the activity of antioxidant enzymes (CAT, SOD, and GR) in mouse lymphocytes, increase intracellular GSH levels, and thereby prevent the formation of intracellular ROS and lipid peroxidation induced by H_2_O_2_ [51,52]. Further research has now found that α-pinene can also inhibit the production of ROS by enhancing the endogenous antioxidant system (GSH levels and the activity and protein expression of CAT, SOD, GR, GPx, and HO-1), thereby reducing lipid peroxidation and regulating cellular redox balance in astrocytes [42]. Although linalool is not the most abundant component in citrus CPEOs, surprisingly, in addition to enhancing the activity of SOD, CAT, and GR, and increasing GSH levels to exert antioxidant protective effects [54], it can further reduce the levels of ROS in mitochondria by maintaining mitochondrial morphology and preserving mitochondrial membrane potential under oxidative stress [55]. Linalool can also enhance antioxidant capacity by increasing intracellular mitochondrial respiration, thereby increasing the energy required to maintain cellular function and cell survival [55]. β-Myrcene, as the main component of *Neocinnamomum caudatum* leaves, has the ability to inhibit the production of intracellular ROS and attenuate the depletion of mitochondrial membrane potential in a concentration-dependent manner [56]. We hypothesized that the antioxidant mechanism of CPEOs might be due to the lipophilic monoterpenes (such as D-limonene, α-pinene, β-myrcene, and linalool), which could penetrate the RAW264.7 cell membrane, enabling them to reach intracellular proteins and targets (including mitochondria). On one hand, these monoterpenes maintained mitochondrial morphology and increased mitochondrial respiration under oxidative stress, thereby enhancing the cellular antioxidant capacity. On the other hand, they increased the expression levels and activities of intracellular antioxidant enzymes, elevated GSH content, and reduced the accumulation of ROS and lipid peroxidation. In this study, JD EO exhibited the highest antioxidant activity among the five CPEOs, which may be attributed to its relatively high contents of D-limonene, α-pinene, and β-myrcene. Although FJ did not have the highest content of D-limonene, it still exhibited relatively excellent antioxidant activity due to its higher levels of myrcene and linalool.

Inflammation is an immune response of organisms to harmful stimuli and is closely related to many progressive diseases [57]. Therefore, preventing or alleviating inflammatory responses can, to a certain extent, prevent the occurrence and further progression of diseases [58]. NO is an important cellular mediator associated with inflammation and is produced by macrophages when stimulated by LPS [59]. The high concentrations of NO produced are mediated by inducible nitric oxide synthase (*iNOS*) and cyclooxygenase-2 (*COX-2*) and may trigger severe inflammatory responses [60]. In our study, all five CPEOs were found to reduce LPS-induced NO production in RAW264.7 cells and increase the inflammatory response. In a similar study, the EO of *Citrus myrtifolia* Raf. inhibited the protein expression of *iNOS* and *COX-2* in LPS-induced RAW264.7 cells, significantly suppressing NO formation [61]. Further evidence has shown that D-limonene and α-pinene in CPEOs can reduce the expression of *iNOS* and *COX-2* in LPS-induced mouse macrophages [62,63]. The anti-inflammatory effects exhibited by the CPEOs in this study may be related to similar mechanisms. Reducing the production of proinflammatory cytokines is considered an effective strategy for improving inflammation-related diseases [59]. Plastina et al. [61] demonstrated that the EO of *C. myrtifolia* Raf. can reduce the production of *IL-6* and *TNF-α* in LPS-induced RAW 264.7 cells. In this study, the five CPEOs decreased the relative expression levels of proinflammatory cytokines (*IL-6*, *TNF-α*, and *IFN-γ*) and the release of proinflammatory cytokines in LPS-activated RAW264.7 cells. The five CPEOs increased the relative expression level of the anti-inflammatory cytokine (*IL-10*) and the release of anti-inflammatory factors in RAW264.7, effectively reducing the LPS-induced inflammatory response from these aspects. This may be because CPEO contains ingredients that effectively inhibit the production of proinflammatory cytokines, such as D-limonene, α-pinene, and β-myrcene, which have been shown to reduce the expression and production of proinflammatory cytokines such as *IL-6*, *TNF-α*, and *IFN-γ* both in vitro and in vivo, thereby alleviating inflammatory responses [62,63,64]. This may be the reason why RA EO has the best anti-inflammatory activity, containing higher levels of D-limonene, α-pinene, and β-myrcene. Additionally, several studies have indicated that the NF-κB and MAPK pathways are closely related to the expression of proinflammatory genes [59]. The expression of *iNOS* is regulated by proinflammatory cytokines [60]. The findings of our study speculate that the anti-inflammatory mechanism of CPEOs may be to regulate the NF-κB and MAPK pathways in RAW264.7 cells activated by LPS. Then, the expression of proinflammatory cytokines was reduced and the expression of anti-inflammatory cytokines was increased. CPEOs thereby reduce the release of proinflammatory cytokines and the content of NO.

The cell cycle progresses from the quiescent phase (G0 phase) to the proliferative phase (G1, S, G2, and M) and then returns to the quiescent phase [65]. During normal cell growth, there is an internal dynamic balance between cell proliferation and apoptosis; however, in glioblastomas, dysregulation of the cell cycle leads to uncontrolled proliferation [66]. Terpenes can prevent the further progression and migration of cancer by engaging in various cellular and molecular activities, such as blocking the cell cycle and inducing apoptosis [67]. In our study, two CPEOs were found to arrest U87 cells in the G0/G1 phase. Similar to the results of this study, the EO of *Lycopus lucidus* Turcz. var. *hirtus* Regel has been shown to arrest Bel-7402 cells in the G0/G1 phase and increase apoptosis [68]. CPEOs can inhibit the proliferation and migration of HepG2 liver cancer cells and HCT116 colon cancer cells, with migration inhibition rates of 55.23% and 49.63%, respectively [69]. Additionally, several studies have shown that terpenes such as D-limonene, α-pinene, and β- myrcene can affect the cell cycle by influencing the expression of cyclin proteins, thereby inhibiting the proliferation of cancer cells [40,42,70]. In this study, CPEOs arrested U87 cells in the G0/G1 phase, leading to apoptosis and preventing the migration and progression of glioblastoma, potentially through a similar mechanism that affects the expression of cyclin proteins.

Apoptosis is a crucial component of the innate tumour suppression mechanism, which inhibits tumourigenesis at multiple stages, from transformation to metastasis [71]. Most chemotherapeutic drugs exert their anticancer effects by regulating apoptosis through modulation of the permeability of the outer mitochondrial membrane [72]. Apoptosis is mediated by either the mitochondrial pathway or the death receptor pathway, with *Bcl-2* family members such as *Bcl-2* and *Bax* serving as key mediators of apoptosis in the mitochondrial pathway [73]. In this study, CPEOs upregulated the mRNA of *Bax* and promoted mitochondrial membrane permeability, indicating the occurrence of apoptosis. Palestine sweet lime EO has been shown to increase the *Bax*/*Bcl-2* protein ratio in colon cancer cells (SW480), activating the endogenous apoptotic pathway [74]. The mechanism by which key components of CPEOs, such as D-limonene, α-pinene, linalool, and β-myrcene, upregulate *BAX* in cancer cells to induce apoptosis has been confirmed in T-cell lymphoma cells, gastric adenocarcinoma cells (AGS), human hepatoma cells (HepG2), human breast cancer cells (MCF-7), and human oral cancer cells (OECM1) [75,76,77]. The apoptotic initiator *caspase-9* is activated due to changes in the MMP caused by *BAX* and further targets and activates the apoptotic effectors *caspase-3* and *caspase-7* [78]. Our qRT-PCR analysis revealed that CPEO treatment significantly increased the gene expression levels of *caspase-9*, *-7*, and *-3*. These genes are well known to be involved primarily in the intrinsic pathway of apoptosis. These findings suggest that CPEOs can induce apoptosis in U87 cells through the mitochondrial apoptotic pathway.

The majority of substances that constitute CPEOs are readily metabolized, with D-limonene being rapidly absorbed in organisms as an example. After orally ingesting D-limonene, rats exhibited rapid intestinal absorption and metabolism. The concentration of D-limonene in the blood rapidly climbed to its peak within 2 h after administration. One hour after oral ingestion, the concentration of D-limonene in the liver reached its peak, and within the subsequent hour, the concentrations of D-limonene in the adrenals and kidneys also peaked [79]. After human ingestion of α-pinene, the concentrations of α-pinene and its metabolites in the blood peak at 1 h and 3 h, respectively. Renal excretion rates increase within 1 to 3 h after oral ingestion, reaching a peak at 3 h. Studies have shown that α-pinene enters the bloodstream through complete intestinal absorption after ingestion and subsequently undergoes rapid and nearly complete hepatic metabolism [80]. Linalool is rapidly absorbed by the human body after oral ingestion. The concentration of linalool in the serum reaches its peak 1.6 h after ingestion, with a related half-life of 3.9 ± 2.9 h [81]. These studies were consistent with the view of this study that CPEO exhibited good bioavailability. A study had confirmed that Eureka lemon peel essential oil by gavage could alleviate liver injury induced by D-galactose in mice. This was achieved through two mechanisms: firstly, it effectively regulated the expression and levels of *Cu/Zn-SOD*, *Mn-SOD*, *CAT*, *HO-1*, *Nrf-2*, *NQO1*, and *GSH-Px* in mice, thereby effectively alleviating hepatic oxidative stress; secondly, it modulated the expression levels and contents of *nNOS*, *iNOS*, *IL-1β*, *COX-2*, *NF-κB*, and *TNF-α*, reducing liver inflammation [82]. This study discovered similar phenomena, indirectly proving that the ingestion of CPEOs had effectively balanced the redox systems in the cells of various organs and reduced the incidence of chronic inflammation. CPEOs not only are directly absorbed and utilized by the human body to exert effects, but their metabolites also exhibit biological activity, jointly participating in regulating body health and function. The metabolism of orally administered D-limonene in organisms occurred through the following four pathways: dihydroxylation of both the inner and outer ring double bonds, oxidation of the methyl side chain, and allylic oxidation of the C6-ring [83]. D-limonene was further oxidized by hepatic cytochrome P450 and enzymes in the kidneys into compounds such as limonene-8,9-diol (LMN-8,9-OH), limonene-1,2-diol (LMN-1,2-OH), perillic acid (PA), perillyl alcohol (POH), and cis- and trans-CAR isomers [84]. The study found that the plasma concentration of PA reached its peak level in humans 1 h after ingestion of D-limonene, with a terminal elimination half-life ranging from 0.82 to 1.84 h [85]. The metabolites of CPEOs, PA, and POH, after undergoing metabolism in the liver and kidneys, re-enter the blood circulation to exert antioxidant, anti-inflammatory, and anticancer effects once again [86,87]. In particular, POH demonstrates a significant inhibitory effect on glioblastoma [87,88]. After oral ingestion, α-pinene undergoes metabolism in the human body, primarily through oxidation of its methyl side chain, resulting in the formation of myristyl alcohol and cis- and trans-verbenol [80]. Verbenol may promote apoptosis in HeLa and MCF7 cells by activating autophagy [89]. In this study, it was found that the anti-U87 cell effect of CPEOs was consistent with this observation. The two CPEOs with the highest content of D-limonene and α-pinene exhibited the strongest inhibitory effect on glioblastoma cells, leading to the hypothesis that CPEOs may exert an anticancer effect through a similar mechanism.

In this study, we investigated the potential applications of citrus essential oils, as a type of natural product with complex compositions and unique chemical properties, in the fields of functional foods and nutritional supplements. Citrus essential oils exist in the form of small molecular compounds, a characteristic that grants them excellent lipid solubility, enabling CPEOs to easily cross cellular membrane barriers and achieve efficient absorption. This high bioavailability is the key distinguishing feature of CPEOs compared to many other antioxidants. In the future, further research should focus on the mechanisms of action of individual active ingredients and conduct mouse trials to fully explore and validate the boundless potential of CPEOs in promoting human health.

## 5. Conclusions

In this study, we found that all five CPEOs had good medicinal and health benefits. Among them, RA and FJ EOs demonstrated superior antioxidant and anti-inflammatory activities, showing good performance in the prevention of chronic diseases. XLB and RA EOs induced apoptosis in U87 glioblastoma cells through the mitochondrial apoptotic pathway, resulting in significant cytotoxicity against U87 cells. Therefore, CPEOs have value as antioxidants, anti-inflammatory agents for health maintenance, and anticancer medicinal substances, making them important candidates for use as natural, nontoxic food additives. The results of this study have important practical significance for further large-scale development of CPEO products and increasing the value of CPEOs.

## Figures and Tables

**Table 1:** Information on *Citrus* materials.

No.	Citrus Resources	Latin Name	Locality (China)	Abbreviation	EO Content (%)
1	XingLuBiXiYou	*Citrus paradisi* Macf.	Zhangzhou, Fujian	XLB	1.01 ± 0.04
2	RongAnJinDan	*Fortunella crassifolia* Swingle cv. Chintan	Rongan, Guangxi	RA	1.56 ± 0.05
3	FengJieQiCheng	*Citrus sinensis* Osbeck cv. Fengjieqicheng	Fengjie, Chongqing	FJ	1.27 ± 0.09
4	JinCheng26	*Citrus sinensis* Osbeck cv. Jincheng26	Jiangjin, Chongqing	JC26	1.19 ± 0.12
5	ShaTangJu	*Citrus reticulata* ’Shatang’	Guilin, Guangxi	STJ	1.33 ± 0.12

**Table 2:** IC_50_ values (µg/mL) of CPEOs in U87 cells.

Five Citrus Essential Oils Semi-Inhibitory Concentration					
U87 IC_50_ (µg/mL)	FJ	JC26	STJ	XLB	RA
	84.81 ± 1.01	89.67 ± 0.87	82.05 ± 0.95	77.57 ± 0.95	81.02 ± 0.93

## Data Availability

All of the data are contained within the article and the Supplementary Materials.

## Supplementary Materials

The following supporting information can be downloaded at: https://www.mdpi.com/article/10.3390/antiox13121562/s1, Figure S1: The effect of mitoxantrone as a positive control on the viability of U87 cells; Table S1: GPS position of the picking location of five citrus materials, the appraiser of the plant material, the specimen number, and the official herbarium; Table S2: Primers used for qRT-PCR of inflammation-related genes in RAW264.7 cells and apoptosis-related genes in U87 cells.
