# Supplementary material for: Antioxidant, Anti-Inflammatory, and Anticancer Activities of Five Citrus Peel Essential Oils
Source: Antioxidants (Basel). 2024 Dec 19;13(12):1562. doi: 10.3390/antiox13121562 (PMC11672981; doi:10.3390/antiox13121562)
Supplement: Supplementary file 1 [file antioxidants-13-01562-s001.zip › antioxidants-3330647-supplementary.pdf]

Figure S1. The effect of mitoxantrone as a positive control on the viability of U87 cells. The IC_{50} value was 16.22±2.49 μg/mL.

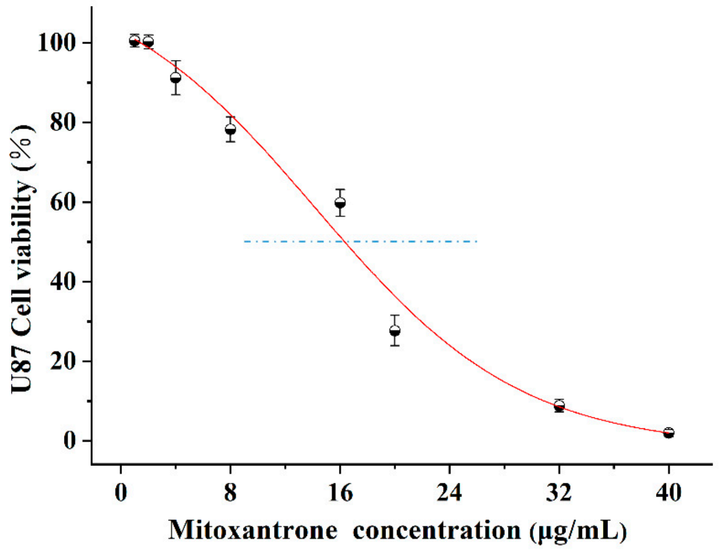

Table S1. GPS position of the picking location of five citrus materials, the appraiser of the plant material, the specimen number, and the official herbarium.

No.	Citrus resources	Longitude	Latitude	Appraiser	Specimen Number	Official Herbarium
1	XLB	117°38′33.0″E	24°30′59.3″N	Zhiqin Zhou	LG0096	Citurs Research Institute, SWU/CAAS
2	RA	109°23′34.2″E	25°13′39.2″N	Zhiqin Zhou	LF0006	Citurs Research Institute, SWU/CAAS
3	FJ	109°24′20.2″E	31°1′23.8″N	Zhiqin Zhou	LS0172	Citurs Research Institute, SWU/CAAS
4	JC26	106°15′38.0″E	29°19′21.7″N	Zhiqin Zhou	LS0040	Citurs Research Institute, SWU/CAAS
5	STJ	110°10′30.3″E	25°14′19.3″N	Zhiqin Zhou	LR0310	Citurs Research Institute, SWU/CAAS

Table S2. Primers used for qRT–PCR of inflammation-related genes in RAW264.7 cells and apoptosis-related genes in U87 cells.

Target Gene	Gene accession number	Primer Sequences (5′–3′) Forward (Fw) and Reverse (Rw)	Length (bp)	Annealing Temperature (°C)	Fragment Size (bp)
GAPDH (Mouse)	NM_008084	F: AGGTCGGTGTGAACGGATTTG<br>R: TGTAGACCATGTAGTTGAGGTCA	21<br>23	62	123
TNF-α (Mouse)	NM_013693	F: CCCTCACACTCAGATCATCTTCT<br>R: GCTACGACGTGGGCTACAG	23<br>19	62	61
IFN-γ (Mouse)	NM_008337	F: ACAGCAAGGCGAAAAAGGATG<br>R: TGGTGGACCACTCGGATGA	21<br>19	62	106
IL-6 (Mouse)	NM_031168	F: TAGTCCTTCCTACCCCAATTTCC<br>R: TTGGTCCTTAGCCACTCCTTC	23<br>21	61	76
IL-10 (Mouse)	NM_010548	F: GCTCTTACTGACTGGCATGAG<br>R: CGCAGCTCTAGGAGCATGTG	21<br>20	62	105
GAPDH (Hum)	NM_001357943	F: GACCTGCCGTCTAGAAAAAC<br>R: TTGAAGTCAGAGGAGACCAC	20<br>20	56	126
Bax (Hum)	NM_138763	F: GGCTATTTCAACCAGGGTTCC<br>R: TGCGAATCACCAATGCTGT	21<br>21	60	155
CASP-9 (Hum)	NM_032996	F: CTCAGACCAGAGATTCGCAAAC<br>R: GCATTTCCCCTCAAACTCTCAA	22<br>22	60	116
CASP-7 (Hum)	NM_004346	F: CGGTCCTCGTTTGTACCGTC<br>R: CGCCCATACCTGTCACTTTATCA	20<br>23	60	175
CASP-3 (Hum)	NM_004346	F: CATGGAAGCGAATCAATGGACT<br>R: CTGTACCAGACCGAGATGTCA	22<br>21	60	139
